# Herd immunity to Newcastle disease virus in poultry by vaccination

**DOI:** 10.1080/03079450701772391

**Published:** 2008-01-16

**Authors:** Michiel van Boven, Annemarie Bouma, Teun H. F. Fabri, Elly Katsma, Leo Hartog, Guus Koch

**Affiliations:** ^1^Animal Sciences Group, Wageningen University and Research Centre, P.O. Box 65, 8200 AB Lelystad, The Netherlands; ^2^Faculty of Veterinary Medicine, Department of Farm Animal Health, Utrecht University, P.O. Box 80151, 3508 TD Utrecht, The Netherlands; ^3^Animal Health Service, Arnsbergstraat 7, P.O. Box 9, 7400 AA Deventer, The Netherlands; ^4^Department of Virology, Central Institute for Animal Disease Control, Wageningen University and Research Centre, P.O. Box 2004, 8203 AA Lelystad, The Netherlands

## Abstract

Newcastle disease is an economically important disease of poultry for which vaccination is applied as a preventive measure in many countries. Nevertheless, outbreaks have been reported in vaccinated populations. This suggests that either the vaccination coverage level is too low or that vaccination does not provide perfect immunity, allowing the virus to spread in partially vaccinated populations. Here we study the requirements of an epidemiologically effective vaccination program against Newcastle disease in poultry, based on data from experimental transmission studies. The transmission studies indicate that vaccinated birds with low or undetectable antibody titres may be protected against disease and mortality but that infection and transmission may still occur. In fact, our quantitative analyses show that Newcastle disease virus is highly transmissible in poultry with low antibody titres. As a consequence, herd immunity can only be achieved if a high proportion of birds (>85%) have a high antibody titre (log_2_ haemagglutination inhibition titre ≥3) after vaccination. We discuss the implications for the control of Newcastle disease in poultry by vaccination.

## Introduction

Newcastle disease (ND) is a highly contagious viral disease affecting wild and domestic avian species (Seal *et al.*, 2000; Alexander, 2003). The impact of ND is most notable in domestic poultry due to the high susceptibility of poultry and the severe consequences of outbreaks of virulent strains on the poultry industries. In fact, it has been argued that ND may represent a bigger drain on the world economy than any other animal viral disease (Alexander, 2003), although the current epizootics of H5N1 avian influenza in Southeast Asia are challenging (if not surpassing) this status.

In response to the threat presented by ND, several countries have put in place vaccination campaigns to prevent epizootics. However, outbreaks have been reported in vaccinated populations despite the fact that vaccination is widely applied (Burridge *et al.*, 1975), as for example in The Netherlands in 1992 to 1993, the UK in 1997, and the USA in 2002 (Alexander, 2003, and references therein).

It is known that vaccination of poultry provides an excellent means to lessen clinical signs of infection caused by virulent Newcastle disease virus (NDV) (Alexander, 2003; Senne *et al.*, 2004; Kapczynski & King, 2005). It has also been known for a long time that vaccination itself (with live vaccines based on non-virulent virus strains) may cause disease and reduced growth in vaccinated birds (Alexander, 2003). As a consequence, there has been a trend to use ever less virulent strains as the seed viruses for vaccine production. Although this strategy has reduced the disease rates after vaccination, it also may have contributed to the fact that current vaccines and vaccination campaigns are not maximally effective in preventing infection and transmission (Burridge *et al.*, 1975; Voeten *et al.*, 1987; Alexander, 2003; Senne *et al.*, 2004; Kapczynski & King, 2005). Hence, it is not clear whether the ultimate goal of prevention of major outbreaks after primary virus introductions can be achieved with current vaccines and vaccination programmes.

Vaccination of large numbers of broiler chickens against ND is usually carried out using non-virulent live virus that is administered by spray or atomist, or via drinking water. These administration techniques usually produce considerable variation in the individual antibody immune responses of vaccinated birds, indicating potential variation in the levels of protection after vaccination (Senne *et al.*, 2004). Therefore, a central question in the control of ND is whether virulent viruses are able to spread in heterogeneously vaccinated populations, and, more specifically, under which conditions (vaccination coverage level, distribution of antibody titres) epidemic spread can be prevented.

To determine whether poultry flocks are at risk of major outbreaks of ND we use the concept of ‘herd immunity’ (Anderson & May, 1991; Diekmann & Heesterbeek, 2000). Epidemiological theory informs us that whether a population is protected against epidemic spread of an infectious agent or not is determined by the reproduction numbers of the infectious agent in populations with high and low levels of protection after vaccination, and by the fraction of animals that have a high level of protection after vaccination. ‘Herd immunity’ is achieved if the fraction of animals with a high level of protection is equal to or greater than a certain critical fraction of animals with a high level of protection. The critical vaccination fraction is in turn determined by the reproduction numbers of the infectious agent in populations with high and low levels of protection (see Materials and Methods for details). The central aim of the present study is therefore to provide estimates of the reproduction numbers of NDV in bird populations with high and low levels of protection after vaccination.

To this end we have carried out an extensive set of experiments with various virus–vaccine combinations. All experiments were performed with broilers that received two vaccine doses, thereby mimicking vaccination schemes in the field. The experiments were carried out with birds that had a low antibody titre (as determined by the haemagglutination inhibition test) after vaccination (log_2_ titre: 0–2), and with birds that had a high antibody titre after vaccination (log_2_ titre ≥3). Throughout we have utilized two commonly used vaccines that are based on the mildly virulent (lentogenic) La Sota and Ulster strains (Alexander, 2003). As challenge virus we used three well-known highly virulent (velogenic) strains: Herts33/56, Netherlands/93, and California/71. On the basis of the data of these experiments we estimated the reproduction numbers of NDV in groups of broilers with low and high antibody titres. We discuss the implications of our findings for the design of vaccines and vaccination campaigns aimed at protection of poultry flocks against major outbreaks of ND.

## Materials and Methods

### Epidemiological considerations

Whether or not a major outbreak can occur after a primary introduction of an infectious agent into a population is determined by the (basic) reproduction number, which gives the expected number of secondary infections caused by one infected individual in a large population in the early stages of an outbreak. If the reproduction number (*R*) exceeds the threshold value of 1 a chain reaction of infection events is possible, and an epidemic can occur. If, on the other hand, the reproduction number remains below the threshold value of one, a major outbreak cannot occur. In a standard compartmental SEIR model (where the compartments represent susceptible, exposed but not yet infectious, infected and infectious, and removed individuals) with one type of infected individual and permanent immunity after infection or vaccination, *R* determines the critical vaccination coverage needed to achieve herd immunity. In fact, epidemiological theory states that herd immunity is obtained if the actual vaccination coverage exceeds the critical vaccination coverage *p*_c_, which is given by *p*_c_ 1 −1/*R* (Anderson & May, 1991).

ND vaccines, however, do not provide unconditional immunity against infection and transmission, and the individual responses to vaccination can be highly variable. As a consequence, we have to adapt the above considerations to allow for variable immunity after vaccination. To keep the analyses manageable, we consider a model with two immune classes: one with a low level of protection against infection and disease, and one with a high level of protection against infection and disease. With two types of individuals, the overall reproduction number depends on the reproduction numbers of the virus in homogeneous populations with high and low levels of protection (*R*_high_ and *R*_low_) as follows:

$$R=pR_{\text{high}}+\left(1-pR_{\text{low}}\right),$$

where *p* is the fraction of individuals that has a high level of protection (Diekmann & Heesterbeek, 2000).

The critical fraction of individuals that needs to have a high level of protection in order for the population to achieve herd immunity, *p*_c_, is given by setting *p* = *p*_c_ and solving the above equation for *R* 1. A straightforward calculation shows that:

(1) $$p_{c}=\frac{R_{\text{low}}-1}{R_{\text{low}}-R_{\text{high}}}.$$

The above theoretical considerations can be refined and extended in a number of directions, but for the present purposes Equation (1) suffices as a rule of thumb specifying the requirements of an epidemiologically effective vaccination programme.

Equation (1) implies that herd immunity cannot be achieved whenever the infectious agent is able to spread epidemically in populations with a high level of protection (i.e. if *R*_high_ > 1), and that no animals need to have a high level of protection whenever the pathogen cannot spread epidemically in populations with a low level of protection (i.e. if *R*_low_ < 1). In the special case that a high level of protection guarantees that no transmission can occur (*R*_high_ = 0) Equation (1) reduces to the familiar *p*_c_ = 1 − 1/*R*_low_.

### Experimental approach

To determine the transmission dynamics of NDV in vaccinated and unvaccinated poultry living in close contact groups, a number of experimental transmission studies with broiler chickens were carried out. All experiments were performed in a high-containment unit under BSL3 + conditions at the Central Institute for Animal Disease Control Lelystad. The experiments complied with the Dutch law on animal experiments and were reviewed by an ethical committee. The design of transmission experiments has been described before (de Jong & Kimman, 1994; Bouma *et al.*, 1996; van der Goot *et al.*, 2005). Briefly, a number of birds that were inoculated with the virus at the start of the experiment were housed with a number of uninfected contact birds. During the course of the experiments the status of the birds (normal, sick, and dead) was recorded, and the serological status of each bird was determined at the start and at the end of the experiments.

Birds were vaccinated at 1 day old by spraying with commercial vaccines—either La Sota (Nobilis® ND clone 30) in Experiments 1 to 3 and Experiments 5 to 7, or Ulster (Poulvac NDW) in Experiments 4 and 8—and received a booster vaccination with the same vaccine using an atomist between 8 and 20 days of age. Birds were transported to animal BSL-3 + facilities of the CIDC-Lelystad laboratory between 22 and 26 days of age, well before the start of the actual challenge experiments. In each experiment 10 birds were inoculated with virus and housed together at day 0. Subsequently, 10 uninfected contact birds were added to the inoculated birds at day 1.

Three different viruses were used for challenge infection (Table 1). For Experiments 1, 4, 5, and 8, a velogenic NDV Netherlands/93 (APMV-1/Netherlands/152608/93) was used. This virus was isolated in The Netherlands during the 1992 to 1993 epizootic from a vaccinated layer flock, and was characterized as a velogenic virus based on the amino acid residues (RRQKR.F) at the cleavage site of the fusion protein. The virus has an intracerebral pathogenicity index (ICPI) of 1.84. Phylogenetic analysis has placed the virus in clade 5a (Aldous *et al.*, 2003) or genotype VII (Lomniczi *et al.*, 1998). In Experiments 2 and 6, Herts33/56 was used as the challenge virus. The Herts33/56 virus used in this study had an ICPI of 1.86, and was obtained from the Veterinary Laboratory Agency (UK) in 1973. Herts33/56 is placed within lineage group 3b (Aldous *et al.*, 2003) or genotype IV (or the separate genotype W) (Lomniczi *et al.*, 1998; Czegledi *et al.*, 2006). In Experiments 3 and 7, a viscerotropic velogenic California/71 virus (Calfornia 2098/71) was used. This virus was isolated form a chicken during the ND outbreak in California in 1971 and was received from Dr Hanson of the University of Wisconsin. This virus has an ICPI of 1.8 and is placed in clade 3c or genotype V. Animals were challenged via the trachea (0.1 ml) and the nasal cavity (0.1 ml) with a suspension containing 10^6^ median egg infectious doses (EID_50_) for Experiments 1 and 5, 10^6^ EID_50_ for Experiments 2 and 6, 10^6.4^ EID_50_ for Experiments 3 and 7, and 10^3.8^ EID_50_ for Experiments 4 and 8.

**Table 1:** Overview of the experimental transmission studies

				Number of contact birds^b^	
				
Experiment	Vaccine	Virus	HI titre^a^ (log_2_)	Infected	Dead
1	La Sota	NL93	0 to 2	9, 10	0, 1
2	La Sota	Herts33/56	0 to 2	5	0
3	La Sota	California71	0 to 2	10	1
4	Ulster	NL93	0 to 2	10, 10	4, 4
5	La Sota	NL93	≥3	0, 4, 10	0, 0, 0
6	La Sota	Herts33/56	≥3	0, 2	0, 0
7	La Sota	California71	≥3	1, 3	0, 1
8	Ulster	NL93	≥3	7	0

Experiments 2, 3, and 8 consist of one trial, Experiments 1, 4, 6, and 7 contain two trials, and Experiment 5 contains three trials. Each trial initially contained 10 infected birds and 10 uninfected contact birds. The vaccines are based on the La Sota (Nobilis Clone30) and Ulster (NDW Poulvac) viruses. The challenge viruses are NL93 (APMV-1/chicken/Netherlands/152608/93), Herts33/56, and California71.

a Range of HI titres of the birds just before the start of the experiment.

b Number of contact birds that were infected or died per trial.

The haemagglutination inhibition (HI) test was performed according to standard EU protocol (EU Council Directive 92/66/EEC). For the test, the amount of Ulster/66 ND antigen was adjusted to 8 haemagglutinating units. Controls on the antigen content in the HI test were carried out using serial two-fold dilutions starting at 1:2, 1:3, 1:4, 1:5, and 1:6. The test was considered valid when the amount of antigen in the control lies between 6 and 9 haemagglutinating units (1 unit being the dilution at which 100% agglutination is still observed). The titre was determined testing two-fold dilution series of sera, and the titre was expressed as the highest dilution showing complete inhibition of agglutination. In each HI test run, a negative control and sera with low, mean and high titre were included. Test results are considered valid only when the titre of these reference sera did not deviate more than one dilution step up or down from the mean.

Throughout we classified birds with log_2_ antibody titres in the range 0 to 2 as having a low level of protection, and birds with log_2_ antibody titres ≥3 as having a high level of protection. This classification scheme was chosen on the basis of earlier evidence, which suggested that log_2_ antibody titres ≥3 were sufficient to protect against disease (data not shown), that log_2_ antibody titres ≥3 are very unlikely to result from non-specific reactions. Moreover, Dutch legislature asks that at least 90% of vaccinated birds of 4 weeks and older have a log_2_ antibody titre of at least 3.

Birds that died with signs of infection or showed an increase of the HI titre of at least two log steps were considered to be infected and infectious after challenge.

### Statistical analyses

The analyses of the transmission experiments follow the line of analyses of earlier transmission experiments with pseudorabies virus in pigs and highly pathogenic avian influenza virus in poultry (de Jong & Kimman, 1994; Bouma *et al.*, 1996; van der Goot *et al.*, 2005). Throughout, the analyses are based on the final size of the transmission experiments; that is, on the number of initially uninfected animals that have been infected during the course of the experiment (Table 1). Here we assume an exponentially distributed infectious period, which gives high estimates of the reproduction number and broad confidence intervals in comparison with models that assume less variation in the infectious period (Ball, 1986). As a consequence, the method also yields a fairly high estimate of the critical fraction of animals that needs to have a high level of protection to obtain herd immunity.

Confidence intervals of the estimated parameters are calculated as reported by Velthuis *et al.* (2007). In short, exact confidence bounds of the parameter estimates are determined by calculating all possible values *r* of the reproduction number *R* for which the hypothesis *H*_0_: *R* = *r* is not rejected; that is, for which the *p* value is larger than 0.05. In the case where all contact animals are infected (Experiments 3 and 4) no estimate of the reproduction number is calculated $\left(\hat{R}\to \infty \right)$, and only the lower bound of the (one-sided) 95% confidence interval is given.

## Results

### NDV infection and disease

All three challenge viruses used in this study are highly virulent as measured by the ICPI (>0.7), and are able to kill experimentally infected, susceptible chickens within 2 days. In our transmission experiments with vaccinated birds, however, only a minority of the birds died during the experiments. In fact, in the experiments with birds that had high antibody titres after vaccination (log_2_ titre ≥3; Experiments 5 to 8), 27 of the 80 contact birds showed signs of infection (Table 1), and just one of these 27 infected contact birds died. In the experiments with birds that had low antibody titres (log_2_ titre: 0–2; Experiments 1 to 4), the majority of the contact birds had signs of infection (54 out of 60), but still only a minority of these infected contact birds died (10 out of 54). Overall, these results indicate that vaccination provides excellent protection against mortality after a natural infection with virulent NDV when vaccinated birds have high antibody titres, and that vaccination is still quite effective in reducing mortality rates in birds with low antibody titres. On the other hand, vaccination does not seem to provide substantial protection against infection, especially in birds having low antibody titres.

### NDV transmission

The data in Table 1 indicates that for most virus vaccine combinations the virus is able to spread extensively in birds with low antibody titres (log_2_ titre: 0–2). In fact, in Experiment 3 (one trial) and Experiment 4 (two trials) all contact birds became infected, and in Experiment 1 (two trials) only one of the 20 contact birds escaped infection. The results of Experiment 2 were somewhat different as five out of 10 contact birds escaped infection.

**Table 2:** Overview of the statistical analyses

Experiments	${\hat{R}}_{low}$	${\hat{R}}_{high}$
1 + 5	3.4 (1.5–8.4)	1.1 (0.46–1.7)
2 + 6	0.95 (0.27–2.8)	0.19 (0.023–0.79)
3 + 7	>1.8^a^	0.38 (0.044–0.98)
4 + 8	>2.1^a^	1.5 (0.50–3.9)
All	3.1 (1.8–4.3)	0.72 (0.41–1.0)

Data are the combined results of Experiments 1 and 5 (vaccine, La Sota; virus, NL93), Experiments 2 and 6 (vaccine, La Sota; virus, Herts33/56), Experiments 3 and 7 (vaccine, La Sota; virus, California71), Experiments 4 and 8 (vaccine, Ulster; virus, NL93), and all experiments taken together. ${\hat{R}}_{low}$ and ${\hat{R}}_{high}$ represent the maximum likelihood estimates of *R_low_* and *R_high_*, which are calculated using the infection data of Table 1. The 95% confidence intervals are presented in parentheses.

a Only the lower bound of the (one-sided) 95% confidence interval is given.

The results of the formal analyses are presented in Table 2. The quantitative analyses of Experiments 1 to 4 yield estimates of the reproduction number *R_low_* varying from ${\hat{R}}_{low}=0.95$ (95% confidence interval: 0.27–2.8) at the low end in Experiment 2 to ${\hat{R}}_{low}>2.1$ with 95% confidence at the high end in Experiment 4. If all transmission experiments with birds that had a low antibody titre at the start of the experiment are combined, we find${\hat{R}}_{low}=3.1$ (95% confidence interval: 1.8–4.3), and we conclude that, in general, NDV is able to spread epidemically in groups of vaccinated broilers with low antibody titres. It should be noted that this conclusion rests on the assumption of no systematic differences in the transmission characteristics of the different viruses. Unfortunately, with the current data this assumption cannot be validated or falsified.

In groups of birds with high antibody titres there was more variation in the outcome of the experiments (Table 1). This is mirrored by the statistical analyses, which show considerable variation in the estimates of the reproduction number *R_high_*: At the low end ${\hat{R}}_{high}=0.19$ (95% confidence interval: 0.023–0.78) in Experiment 6 to ${\hat{R}}_{high}=1.5$ (95% confidence interval: 0.50–3.9) at the high end in Experiment 8. Notice that all experiments indicate that at least some spread is possible in vaccinated birds with high antibody titres, and that Experiments 5 and 8 do not exclude the possibility of epidemic spread in populations with high antibody titres (i.e. *R*_high_ > 1): If, however, we assume that differences between different sets of experiments are the result of chance and combine all experiments with birds having high antibody titres, the estimate of the reproduction number is ${\hat{R}}_{high}=0.72$ (95% confidence interval: 0.41–1.0), and we conclude that epidemic spread is unlikely in groups of birds having high antibody titres.

### Herd immunity against NDV by vaccination

Herd immunity to NDV is obtained whenever the virus is unable to cause a prolonged chain of infections; that is, if no epidemic can unfold after a primary virus introduction (Diekmann & Heesterbeek, 2000). In our context, ‘herd immunity’ is achieved if the fraction of birds that have a high antibody titre after vaccination exceeds the critical fraction of birds with a high antibody titre specified by Equation (1). For the combined analysis of the experiments (Table 2) and assuming that 100% of the birds are vaccinated, the critical fraction is estimated at *p*_c_ = 0.88 (i.e. at least 88% of the birds need to have a high antibody titre after vaccination to prevent a major epidemic). We would like to stress that there is considerable uncertainty surrounding this estimate. For instance, if we take the lower and upper bounds of the confidence intervals of ${\hat{R}}_{low}$ and ${\hat{R}}_{high}$, and insert these values in Equation (1), we obtain *p*_c_ = 0.58 and *p*_c_ = 1 as lower and upper bounds of the critical fraction of birds with high level of protection. In other words, *p*_c_ may be as low as 58% or as high as 100%. It should be noted, however, that these bounds are conservative in view of our estimation method that yields broad confidence intervals for the reproduction numbers (see Materials and Methods).

Also shown in Table 2 is substantial variation in the estimates of reproduction numbers of the individual experiments. As a consequence, there is also considerable variation in the estimates of the critical vaccination fraction if we focus on experiments with identical virus–vaccine combinations (Experiments 1 + 5, Experiments 2 + 6, Experiments 3 + 7, Experiments 4 + 8). For Experiments 2 + 6 the critical vaccination fraction is estimated to be 0%, indicating that epidemic spread of virus is unlikely even in vaccinated populations of birds with low antibody titres $\left({\hat{R}}_{low}<1\right)$ For Experiments 3 + 7 the lower bound of the estimate of the critical vaccination fraction is 46%, and for Experiments 1 + 5 and Experiments 4 + 8 herd immunity cannot be achieved even if all birds have a high antibody titre after vaccination since the virus is expected to able to spread epidemically even in populations with high antibody titres $\left({\hat{R}}_{high}>1\right)$.

## Discussion

In this paper we have presented quantitative analyses of NDV transmission in vaccinated chickens. Our results indicate that although vaccination in general provides good protection against disease and mortality, it may not provide sufficient protection against virus transmission so as to be able to prevent or halt epidemics of ND. This finding is of considerable interest as it brings into question the epidemiological effectiveness of current vaccination campaigns against ND. Overall, our analyses indicate that a high fraction of birds (>85%) needs to have a high antibody titre (log_2_ titre ≥3) after vaccination to ensure that no epidemic spread is possible in vaccinated populations.

In view of our results, a central question is whether it is possible to obtain consistently high antibody titres using the current administration techniques (by spraying or via the drinking water) of ND vaccines based on viruses of low virulence. Unfortunately, to date there are no systematic studies that have investigated the distribution of antibody titres after vaccination of large populations of poultry. A pilot experiment in The Netherlands suggests that it may be possible to obtain high antibody titres in the majority of birds, but only if strict preconditions on the vaccine content and administration techniques are met (data not shown). It should also be noted that in the absence of circulation of virulent virus in a region there may be an incentive for farmers to use vaccination schemes and procedures that are not epidemiologically optimal because of the negative side-effects of vaccination.

An additional issue that needs attention is the possibility that the virus might spread unnoticed in partially vaccinated populations where it would be detected quickly in unvaccinated populations, thereby increasing the period in which the virus can be transmitted unchecked. Whether this theoretical possibility might become a real problem is not known at present. Certainly, our results and those of Kapczynski and King (2005) have shown that infection, shedding, and transmission of virulent NDV in vaccinated birds may occur without overt disease signs. Given this possibility we believe that, if preventive vaccination programmes are to be implemented, they should go together with a monitoring programme ensuring that sufficient flock immunity levels are achieved. Similar views have recently been expressed for highly pathogenic avian influenza viruses in poultry (Capua & Marangon 2006; Capua & Alexander, 2006; Savill *et al.*, 2006).

Our results have shown some variation in the transmission levels using different combinations of vaccine and virus (especially in groups of birds with a high antibody titre). On the one hand, in view of the limited amount of data for each particular virus-vaccine combination, it may be that the variation in transmission is a result of chance. On the other hand, it may also be that our data have revealed a trend that hints at variable efficacy of different vaccines against different viruses (see also Kapczynski & King, 2005). In this respect, it is of note that better protection appears to be obtained against challenge viruses that were isolated in the 1930s and 1970s (i.e. Herts33/56 and California71), and which are genetically more closely related to current vaccine strains than the more recently isolated and genetically more distinct Netherlands/93 virus (Lomniczi *et al.*, 1998). This is in line with recent results (Czegledi *et al.*, 2006), which indicated that current vaccines induced better protection against viruses that were isolated in past epizootics than against viruses that are currently circulating. With this in mind, it would be interesting to investigate the antigenic relationship between past and current circulating viruses, and the effect of the antigenic match between virus and vaccine on the level of protection conferred against disease, shedding, and transmission. From a practical perspective, since the effectiveness vaccines is ultimately determined by their ability to curb or halt epizootics of ND, we believe that vaccine development should be focused on providing vaccines that protect against infection and shedding rather than against disease.
